# Meeting of the American Medical Association

**Published:** 1856-06

**Authors:** 


### NINTH ANNUAL MEETING OF THE AMERICAN MEDICAL ASSOCIATION, HELD AT DETROIT, MICHIGAN.

Morning Session.—This Society began its session May 6th, at 11 o'clock, at Fireman's Hall. Present, Dr. Geo. B. Wood, of Penn., President; Dr. Daniel Tilden, of Sandusky, Ohio, Vice President; Dr. Brodie, of Detroit, Secretary.

Dr. Pitcher arose and begged leave to say a few words before the regular order of business should be taken up, and proceeded as follows:

Mr. President:—In the name of the Physicians of Michigan, who are here represented by delegates from their state district and more local societies, we welcome the members of the American Medical Association, to the State and City of our adoption.

As children who have wandered from their family altar, to improve their fortunes in new and distant lands, would meet with bounding hearts, the patriarch of their early home, so we, whose fortunes have been cast in the forests of the west, now greet with kindred emotions, the delegates from the old Colonial States, hallowed in their memory by their revolutionary association, honored for the elegance and durability of their institutions of learning, and cherished as the home and birthplace of many of the most brilliant and highly cultivated intellects in our national domain.

With a fraternal attachment no less ardent, we receive the members coming from other States of the confederacy, which like our own, have a position among the other States of the Union, but by the accident of birth are excluded from a place among the stripes of our national escutcheon.

And to our brethren who are here by invitation from the British Provinces in America, with whom from a common ancestry we have derived by inheritance our best and earliest ideas of civil liberty, much of our literature, and many of the practical precepts which requite our art, we offer a like and cordial reception.

Although actively engaged in the battle of life, and earnestly struggling to overcome the obstacles which in our undeveloped country lie in the way of professional success, we have striven like the devoted Parsee, to keep alive the fire which in our youth we kindled at the altar of those Magii, who now come not like the wise men of the east, under the guidance of a merely risen star, by acts of devotion to celebrate the advent of a Messiah, but to receive from us on this ground from which the foot prints of the savage have scarcely been erased by the ploughshare of the white man, when the boat song of the lively Gascon, may still be heard between the strokes of the paddle wheel and the whistlings of the locomotive, the tokens of a sincere friendship, the acknowledgment of a legitimate paternity and the homage due from filial and grateful hearts.

The student of our political history is well aware that under the pressure of exterior force, we have been compelled, on five different occasions, to change our national colors, but never to abjure the faith of our political sires;—so we now intend steadfastly to stand by the true in medicine under all forms of temptation as we will under all the phases of political fanaticism defend the Ark of the Covenant of our political fathers.

We pray that the meeting of this Association, though purely scientific in its aims, may be so conducted as to become instrumental in promoting these great ends.

Again, gentlemen, I bid you, from whatever land, or State, or section of country you may have come, in the name of common brotherhood in science, a warm and cordial welcome.

An intermission of fifteen minutes was then had, to give the delegates for the several States an opportunity to appoint one from each delegation, to act as a nominating committee, to report nominees for the offices of the Society for the ensuing year.

The following constituted the nominating committee:

Maine—N. P. Monroe; New Hampshire—H. Peirce; Vermont—C. L. Allen; Massachusetts—H. H. Childs; Rhode Island—J. E. Warren; Connecticut—David Harrison; New York—William Rockwell; New Jersey—L. A. Smith; Pennsylvania—John Neill; Delaware—J. W. Thomson; Maryland—P. Wroth; South Carolina—E. Geddings; Tennessee—J. B. Lindsley; Kentucky—W. L. Sutton; Minnesota—C. W. Le Boutillier; Michigan—M. Gunn; Ohio—Thos. W. Gordon; Indiana—Dr. Winton; Illinois—H. Noble; Wisconsin—W. H. Brisbane; U. S. Army—Chas. Tripler; Iowa—Dr. McGugin.

The Committee retired to transact their business, and Dr. Pitcher from the Committee on arrangement reported that the regular hours of the Session would be as follows:

Tuesday, from 9 A. M. to 12½ P. M. Afternoon from 2 to 5 o'clock.

Wednesday, 9 A. M. to 12½ P. M., and no afternoon Session, in consequence of a pleasure excursion, to be given on board the Michigan C. R. R. Co.'s steamer, Western World.

Thursday, 9 A. M. to 12½ P. M. Afternoon, 2 to 5 o'clock.

Friday, to begin at 9 A. M. and adjourn at the pleasure of the Society.

The President announced that he had received a communication from Boston, stating that Dr. J. C. Warren, of Boston, one of the Vice Presidents, died on Saturday last.

On motion, the convention adjourned to 2 P. M.

Afternoon Session.—The Secretary read a letter from Dr. Grafton Tyler, of District of Columbia, one of the Vice Presidents, expressing regret at not being able to attend this Convention.

He also read an invitation to this Association from the Tennessee Medical Society, and also from individual physicians of Nashville, tendering the hospitalities of that city, and the use of the Legislative Hall, for the sessions of the Association, to hold its next meeting at that city. Referred to the committee on nominations.

The committee on nominations reported that they had chosen the following officers for the ensuing year:

President: ZINA PITCHER, of Detroit. Vice Presidents: T. W. Blatchford, N. Y.; W. H. Boling, Ala.; E. Geddings, S. C., and W. H. Brisbane, Wis. Secretaries: Wm. Brodie, Mich. and W. C. Foster, Tenn. Treasurer: Casper Wister, Penn. Confirmed.

Second Day.—The Association was called to order by the President, at nine o'clock, A. M.

The minutes were read, corrected and approved.

Dr. Wister, of Pa., read the list of delegates who had registered their names since the last report.

The Secretary read communications from the following gentlemen, asking an extension of time in which to report upon the subjects named:

Dr. A. J. Semmes, of N. Y.—"Coroners' Inquests."

Dr. J. Taylor Bradford, of Ky.—"Treatment of Cholera."

Dr. J. M. Reese, of N. Y.—"Infant Mortality."

Dr. E. R. Peaslee, of Me.—"Inflammation, etc."

Dr. J. W. Corson, of N. Y.—"The Causes of the Impulse of the Heart, and the Agencies which Influence it in Health and Disease."

Dr. Mark Stephenson, of N. Y.—"The Treatment Best Adapted to each Variety of Cataract, with the Method of Operation, Place of Election, Time, Age, etc.

Dr. Beech, of Mich.—"Medical Topography, and Epidemics."

Dr. J. C. Hutchinson, of N. Y.—"The Anatomy and Histology of the Cervix Uteri."

Referred to Committee on Nominations.

The Secretary announced that he had received the following resolution, adopted at the last meeting of the New York State Medical Society:

*Resolved*, That the members of the American Medical Association be invited to attend the semi-centennial celebration of this society, which will occur on the first Tuesday of February, 1857.

The invitation was accepted.

The Secretary read the following communication, dated April 7, 1856, from the Secretary of the Ohio State Medical Society.

Sir—At the annual meeting of this society, held in June last, at Zanesville, Ohio, the following resolutions were adopted, and I was directed to furnish you with a copy of the same:

*Resolved*, That the resolution offered by Dr. Grant (a member of this society, but not at this or that time a practitioner of medicine, but a lawyer,) at the last session of this society, viz: "That it is not derogatory to medical dignity, or inconsistent with medical honor, for medical gentlemen to take out a patent right for surgical or mechanical instruments," was offered at a time when many members had left for their homes, and is not, therefore, the sense of the society.

*Resolved*, That the said resolution is in direct opposition to the code of medical ethics adopted by this society; and, therefore, be it further

*Resolved*, That said resolution, offered by Dr. Grant, and adopted by the society, be and is hereby, rescinded.

The communication was ordered to be placed upon the minutes.

The Secretary read a communication from Dr. Hamilton, of Buffalo, N. Y., transmitting the second part of a report upon deformities after fracture and dislocations, and asking for a correction of the minutes of last session in regard thereto. Dr. H. also asked that he be permitted to incorporate, in a volume upon the subject he is preparing for publication, that portion of the report already published by the Association.

On motion of Dr. Brodie, of Mich., the minutes were amended.

Dr. Atlee, of Pa., offered a resolution that the request of Dr. H., in regard to the publication of the report, be granted.

Dr. Lindsley, of Tenn., opposed the resolution. A similar request was denied at the session of the Association held at St. Louis.

Dr. Palmer, of Ill., moved to refer the matter to a special committee. Carried.

The President appointed as such committee, Drs. Palmer, of Ill., Atlee, of Pa., and Hills, of Ohio.

Dr. Gunn, of Michigan, moved that those gentlemen from Canada, who are here by general invitation, be admitted in a body, and be requested to take seats on the platform during this morning's session. Carried.

The following gentlemen complied with the invitation:

Dr. E. M. Hodder, F. R. S. Eng., Professor of Midwifery and Diseases of Children, Trinity College, Toronto.

Dr. J. H. Richardson, M. R. C. S. Eng., Examiner in Anatomy, University of Toronto.

Dr. Norman Bethune, M. R. C. S. Eng., Prof. of Anatomy, Trinity College, Toronto, C. W.

Dr. Worthy Haswell, M. R. C. of Surgery, Eng.

Dr. A. K. Dewson, College Physicians and Surgeons, New York, Licentiate of Province of the Canadas.

Dr. Geo. Coatsworth, Medical Department University of Buffalo, Licentiate of Province of the Canadas.

Dr. John Tarquand—Woodstock, C. W.

In receiving them upon the platform, the President, Dr. Pitcher, said he was happy to be the instrument of celebrating the nuptials by which we effect a scientific re-union of the two members of the Anglo-Saxon race, which have so long been separated by the political relations having their origin in the separation of the American colonies from the English crown.

Dr. Hodder, in behalf of his Canadian brethren, thanked the Association for the courtesy and kindness extended to them.

Dr. Sutton, of Ky., offered a resolution that 1,000 copies of the address of the late President, Dr. Wood, be published. Adopted.

Dr. Gross, of Ky., read a report on "The Causes which Impede the Progress of American Medical Literature. In conclusion, he submitted ths following resolutions:

*Resolved*, That this Association earnestly and respectfully recommends: 1st. The universal adoption, whenever practicable, by our schools, of American works, as text-books for their pupils. 2d. The discontinuance of the practice of editing foreign writings. 3d. A more independent course of the medical periodical press towards foreign productions, and a more liberal one towards American; and, 4th. A better and more efficient employment of the facts which are continually furnished by our public institutions, for the elucidation of the nature of diseases and accidents, and, indirectly for the formation of an original, a vigorous, and an independent national medical literature.

*Resolved*, That we venerate the writings of the great medical men, past and present, of our country, and that we consider them as an important element of our national medical literature.

*Resolved*, That we shall always hail with pleasure any useful or valuable work emanating from the European press, and that we shall always extend to them a cordial welcome, as books of reference, to acquaint us with the progress of legitimate medicine abroad, and to enlighten us in regard to any new facts of which they may be the repositories.

Dr. Phelps, of New York, moved that the report and resolutions, as a whole, be adopted.

At the suggestion of a member, the question was divided. The report was adopted.

Upon the reading of the first resolution, a member proposed to substitute "just" for "liberal" in line 7. Dr. Gross accepted the amendment.

Dr. Palmer, of Ill., wished to understand the meaning of the word "practicable," as employed in the resolution, (line 3.) If it meant that an inferior work by an American author was to be used in our medical schools in preference to a superior one, treating of the same subject, by a foreign author, he was decidedly opposed to the resolution. If, when the American work is equal or superior to the foreign one, it is to be used, then he had no objection. He alluded to works by eminent English and French authors.

Dr. Gross explained. One of the works alluded to by Dr. P. must of necessity be used in our medical institutions of learning, as there is no work, by an American author, on the same subject. Foreign works should be used as books of reference, and American books, "when practicable," as text books. Dr. Yandel, of Ky., moved that the resolutions be made the special order for Thursday morning. Lost.

Dr. Cobb, of N. Y., was opposed to the resolutions. If adopted and sent out to the world, they savor too much of know-nothingism to make them palatable. [Sensation.]

Dr. Leide, of Pa., was in favor of leaving to teachers of medicine the selection of their own text books.

Dr. Davis understood there was another report touching upon the subject—that upon "American Medical Literature," by Dr. Breckenridge, of Ky. He moved to lay the resolutions upon the table until that report was read. Carried.

The Secretary read a communication from Dr. P. A. Jewett, of Conn., chairman of the Committee to procure memoirs of the eminent and worthy dead. Referred to the Committee on nominations.

Dr. Breckenridge, of Ky., read a report upon American Medical Literature.

On motion of Dr. Hooker, it was accepted and referred to the committee on publication.

The Association then adjourned.

THIRD DAY—MORNING SESSION.—The President called the meeting to order. The Secretary read the minutes of yesterday's session, which were amended and approved.

Dr. McGugin, of Iowa, was chosen a member of the Committee on Nominations to represent that State.

Dr. Atlee, of Pa., offered a resolution that the President have authority to appoint annually delegates from gentlemen going abroad to represent this association in the meetings of the British Medical Society at Paris, and such other foreign bodies as may be in existence. Carried.

The Secretary read letters from the following gentlemen, on the following subjects: From Dr. P. Wroth, of Md., submitting a report from the committee on "The Medical Topography and Epidemics of the United States," on the Medical Topography of the eastern section of Maryland, and asking an extension of time.

From Dr. E. S. Lamoine, of St. Louis, transmitting an acknowledgement of the reception of the proceedings of the association by the Paris Academy of Medicine.

From Dr. D. Thompson, of Louisville, Ky., asking for an extension of the time to report on "The Remedial Effects of Chloroform." All referred to the Committee on Nominations.

From the Secretary of the Michigan State Agricultural Society, transmitting 25 copies of its transactions of 1853 and '54. This was accepted and ordered to be distributed one in each State as far as they would go, and the thanks of the association were returned therefor.

Dr. Clendennin, of Ohio, offered a resolution that a committee of one be appointed to continue three years to report on the Etiology and Pathology of Epidemic Cholera in the United States," and that he have authority to select such other members to assist him as he may deem desirable. Referred to Committee on Nominations.

Dr. Mendenhall, of Ohio, offered a resolution expelling from the association Dr. C. H. Cleveland, of the Cincinnati Eclectic Medical Institute. Carried.

Dr. Atlee, of Pa., offered a resolution that Dr. James R. McClintock, whom he denounced as an "advertising quack," be also expelled. Carried.

Dr. Bloodgood, of Chicago, offered a resolution that the constitution be so amended as to provide that the President shall be elected by ballot and it shall not be requisite that he be a resident of the State wherein the association may be held. Laid over one year under the rule.

Dr. Gunn stated that many of the eastern members desired to return home on the Western World. He had consulted the agent of the line, who had volunteered to start the boat for Buffalo Friday noon, in time to reach that city for the early morning train, providing 50 members would signify their desire to take the boat at that time.

On motion of Dr. Palmer, of Chicago, Dr. Mussey, of Cincinnati, Ex-President of the Society, and Bishop McCroskey, of Detroit, were invited to take seats upon the stand.

The President welcomed the Bishop by stating that not only was he "High Church" in religion, but "High Church" in medicine. He descended from legitimate medical stock.

Dr. Gluck, of New York, moved that the association appoint four delegates to the Medical Congress to be held this season in Europe; these delegates to be appointed from the four sections of the Union. Laid on the table.

Dr. Stocke, of Philadelphia, offered an amendment to the constitution, whereby the association shall meet triennially at some place to be designated hereafter, as a permanent place of meeting; the intermediate year the association shall meet at any place that may be fixed upon; that one permanent Secretary and two assistants be chosen; that the permanent Secretary shall keep the archives of the association in the place of its permanent meeting, and his traveling expenses in going to and from the place of permanent meeting be allowed him.

Dr. Watson, of New York, opposed the amendment.

Dr. Dorsey, of S. C., offered an amendment to the constitution, that the Association meet triennially at Washington, to begin in 1858, and that the Secretary correspond with the Managers of the Smithsonian Institute, to arrange a plan whereby the archives of the Association may be kept in that Institution. Both amendments laid over one year.

Dr. Sheets offered a resolution, that it is derogatory to the profession to notice in our medical journals the works or writings of irregular practitioners. Adopted.

Dr. Wistar, of Pa., offered a resolution that the invitation of the medical gentlemen from Canada to attend the next meeting of the Association, be renewed for the next meeting of the Association at Nashville; and that such of the Canadian profession as can be present at that meeting, are requested to obtain letters of introduction from Drs. Tarquand, A. Scott, of

Woodstock, Drs. Harswell, Widmer, Beaumon and Hewich, of Toronto; Drs. O'Rielly, Craigie and Duggan, of Hamilton; Dr. Sampson, of Kingston. Carried.

When the call upon the Committee on the Medical Topography and Epidemic Diseases of the United States came up, Dr. Thompson, of Delaware, Chairman of that Committee, appointed at its meeting the past summer at Newport, with Joseph Mauran, M. D., as Secretary, remarked that he would detain the Association but a few moments, as there were some explanations connected with this important subject due the Association, to other individuals and himself. The first was, that it was now universally conceded that the period of three years was granted by the Association to the associates of the States and Territories to complete their work; secondly, that in the rearrangement of this important undertaking the power was conceded to each State and Territory to appoint its own historian—which was afterwards confirmed by the Association. It was alone intended to ensure success by imposing the duty upon them as a State right, and not to report upon past committees appointed at Charleston, and given three or four States to take charge of, though to the honor of several of the Western and South-Western States — he said some reports, in whole and in part, had been already published in the transactions of the past year that would scarcely be surpassed by those that should come after them. In the substitution of the new arrangements calculated to insure the completion of this work, and so largely acquiesced in by the Association, he begged leave here, in justice to himself and others, to say that he had, and could not have had, any personal feeling—or preferences, as to appointments, as they alone were made by their own representatives—and these explanations were due both to the old Committees and himself.

Dr. Palmer, from the Special Committee, appointed to receive and consider the request of Dr. F. H. Hamilton, of Buffalo, for permission to use such portion of his report on "Deformities after Fracture," as he may deem proper in his forthcoming book, reported in favor of granting the request, providing the book should not be issued until the report which he may further make, shall have been published by the Association. Carried.

The report and resolution of Dr. Gross, read yesterday, were taken up, discussed for a few moments, and referred to the Committee on Publication.

The Secretary read a letter from Dr. L. H. Steiner, of Washington, transmitting his report on "Strychnia, its Chemical and

Toxicological properties," and regretting his inability to be present in person. Referred to Committee on Publication.

Dr. Palmer, of Chicago, from the Special Committee, read a report on "The Plan of Organization of State and County Societies." It closed with resolutions that the National Association call upon all medical men everywhere to organize themselves into State and County societies; that the Association recommend to all local and county societies to incorporate into their by-laws a provision whereby a board of censors shall be appointed to examine all applicants for membership before they shall be admitted—that members of these societies be urged to make at least brief records of all cases arising in their practice from general or epidemic causes, to report these to the society to which they belong, or to a committee of the society, and that a committee arrange and collect these reports and transmit them to the State Society, to which they may and shall be auxiliary, and the State committee shall arrange and collate these reports, transmit them to the National Association—that the National committee receive these records transmitted from the several States, and collate and prepare them for publication in the transactions—that the code of medical ethics adopted by the National Association be recommended to the local and county societies for adoption—and that it be recommended to the several local and county societies to report to the State society to which they may be auxiliary, such papers presented to them as may be deemed worthy of preservation in a permanent form, thus stimulating to the production of valuable records and papers.

The report, after a brief discussion, was referred to the Committee of Publication and the resolution temporarily laid on the table.

Dr. Davis, of Illinois, read a report from the Special Committee on "The Changes in the Composition and Properties of the milk of the human female, produced by Menstruation and Pregnancy." This was a very scientific and interesting paper, showing very close examination and diligent inquiry and study, with the record of many actual experiments performed by the chairman of the committee. Referred to the Committee on Publication.

The Secretary read a letter from Dr. Charles Chandler, of Rocheport, Mo., who was appointed to report on "Malignant Periodic Fevers," transmitting a paper instead on "Sulphat of Cinchona." Accepted and referred to a special committtee consisting of doctors.

The report on "the excretions as an index to the organic changes going on in the system," by Dr. H. A. Johnson, of Chicago, was called for.

Dr. Davis, of Ill., said he had been requested by Dr. J. to ask for an extension, the report having been already continued for one year. He was opposed to such continuances. If men want to work let them do so; if not, don't let them undertake it. The subject of continuances was referred to the Committee on Nominations.

Several other reports were called for, but their authors were not present.

Dr. Jas. M. Newman, of Buffalo, read an abstract of a lengthy report on "The Sanitary Police of Cities"—a very valuable paper, containing the result of a great variety of examinations, comparisons, etc. Referred to the Committee on Nominations.

The Convention here adjourned till 2 P. M.

THIRD DAY—AFTERNOON SESSION.—The President called the Association to order at 2 P. M.

Dr. Frost, Charleston, S. C., offered a resolution of thanks to the Retiring Officers of the Association and the Committee on Publications. Carried.

Dr. A. J. Fuller, of Maine, read a report on "The Best Treatment of Cholera Infantum." Referred to Committee on Publication.

Dr. Horace Green, of N. Y., read an extract of the report on "The Use and Effect of the application of Nitrate of Silver to Diseases of the Throat." Referred to Committee on Publication.

D. J. B. Flint, of Louisville, Ky., read a report on "The best mode of Rendering the Medical Patronage of the national government tributary to the Honor and Improvement of the Profession. Referred to the Committee on Publication.

"SPECIAL COMMITTEES."—The Committee on Nominations reported the following chairmen on special reports for 1857:

Dr. E. R. Peaslee, of Brunswick, Me., on "Inflammation—its Pathology and its relation to the Recuperative Process."

Dr. J. C. Hutchinson, of Brooklyn, New York, and Chas. E. Isaacs, of New York, on "The Anatomy and Histology of the Cervix Uteri."

Dr. J. Taylor Bradford, of Augusta, Ky., on "The Treatment of Cholera."

Dr. Mark Stephenson, of N. Y., on "The Treatment best adapted to each variety of Cataract with the Method of Operation, place of Election, Time, Age, etc.

Dr. J. W. Carson, of N. Y., on the "Causes of the Impulse of the Heart and the Agencies which influence it in health and disease."

Dr. D. Meredith Reese, of New York, on "The Cause of

Infant Mortality in large cities, the Source of its increase, and the means for its diminution."

Dr. J. Foster Jenkins, of Yonkers, N. Y., on "Spontaneous Umbilical Hemorrhage of the newly born."

Dr. Henry Carpenter, of Lancaster, Pa., on "The Use of Instruments in Obstetrical Practice."

Dr. Alexander J. Semmes, Washington, D. C., on "The Measures to be adopted to remedy the evils existing in the present mode of holding Coroner's Inquests."

Dr. J. Marion Sims, of New York, on "The Treatment of the Results of Obstructed Labor."

Dr. J. B. Flint, of Louisville, Ky., on "The True Position and Value of Operative Surgery as a Therapeutic Agent."

Dr. G. Volney Dorsey, of Piqua, Ohio, on "The causes and cure of Indigestion, especially in relation to the Therapeutic Indications to be derived from the chemical composition of the deposits in the urine."

Dr. C. P. Coventry, of Utica, N. Y., on "The Medical Jurispudence of Insanity and the testimony of Skilled Witnesses in Courts of Justice."

Dr. Joseph Leidy, of Philadelphia, Pa., on "Human, Animal and Vegetable Parasites."

Dr. M. D. Darnally, of Bainbridge, Indiana, on "The value of a strict attention to position in the treatment of diseases of the Abdomen."

Dr. G. Sutton, of Aurora, Indiana, on "Milk Sickness."

Dr. Clark G. Pease, of Jonesville, Wis., on "The blending and conversion of the Types of Fever."

Dr. B. S. Woodworth, of Indiana, on "The best substitute for Cinchona and its preparations in the treatment of Intermittent Fever and Malarious Neuralgia."

Dr. Franklin Hinkle, of Marietta, Pa., on "The use of Cinchona in Malarious Diseases."

Dr. Henry J. Campbell, of Augusta, Ga., on "The Nervous System in Fevrile Diseases." Dr. John Kill, of Phil., in "The Laws governing the Deposit of Bone." Dr .John W. Greene, of N. Y., in "The intimate effects of certain Toxicological agents in the Animal Tissues and Fluids."

Dr. Geo. Suckley, U. S. A., on "The Medical Topograyhy and Fauna of Washington Territory."

Dr. James Cooper, of Hoboken, N. J., on "The Flora of Washington and Oregon Territories." Dr. Chas. E. Isaacs, of N. Y., on "The Intimate Structure and the Pathology of the "Kidney." Dr. Israel Morse, of N. Y., on "The Diseases incidental to Europeans from Temperate Climates in their transition through Central America."

Standing Committees.—The Committee on nominations, also reported the following Standing Committees in the year 1857:

*On Medical Education:*—Drs. S. W. Butler, of N. J., Chairman; Alonzo Clark, N. Y.; C. W. Le Bentillier, of Minnesota; G. F. Mitchell, of Ohio; S. W. Clinton, of Alabama.

*On Medical Literature:*—Dr. R. Hill, of Ohio, Chairman; E. W. Yandell, of Ky.; R. R. Porter, of Del.; H. A. Johnson, of Ill.; Chas. E. Swan, of Maine.

*On Publications:*—Drs. Francis G. Smith, of Pa., Chairman; Caspar Wistar, Pa.; Samuel L. Hollingsworth, of Pa.; Samuel Lewis, of Pa.; H. T. Asken, of Del.; Wm. Brodie, of Mich.; R. C. Foster, of Tenn.

*On Prize Essays:*—Drs. Wm. K. Bowling, Chairman; E. B. Haskins, Thos. Lipscomb, A. H. Buchanan, R. W. Avent, W. A. Cheatham, Paul F. Eve, all of Tennessee.

*On Arrangements:*—Drs. C. K. Winston, Chairman; Iro Connell, Wm. D. Haggart, J. L. C. Johnson, F. A. Ramsay, Geo. Grant, J. B. Lindsley, all of Tennessee.

The Committee on nominations reported the following gentlemen to fill vacancies on Committees.

On "Medical Topography and Epidemics:" Dr. F. P. Fitch, of Amherst, N. H.; Dr. Robt. Murray, of Fort Miller, Cal.

On "The Registration of Births, etc:" Dr. Wm. B. Casey, of Middletown, Conn.; Dr. R. W. Haxall, of Richmond, Va.; Dr. Arthur R. Stout, of San Francisco, Cal.

The Committee recommended the continuance of the Committee to procure memorials of the eminent and worthy dead and the report as prepared be referred to the Committee on Publications.

Fourth Day—Morning Session.—The President called the Association to order. The Secretary read the minutes of the preceding Session, which were corrected and approved.

Dr. Palmer, of Illinois, moved that Dr. Richard Coolidge, of D. C. be substituted in place of Dr. Finley, on the Committee to report on Epidemics. Carried.

Dr. Gunn, of Detroit, from Committee on Credentials, reported the following new members, by invitation:

Dr. Landon, of Monroe; introduced by Dr. Rice.

Dr. R. K. Rodgers, Suspension Bridge, N. Y.; introduced by Dr. Brodie.

Dr. Dwight Nimms, of Calhoun co.; introduced by Dr. Gunn. Adopted.

Dr. Atlee, of Pa.; offered the following:

*Resolved*, That all voluntary communications hereafter presented to the Association, shall be referred to a special committee of —— to be appointed by the President, on the first annual meeting, whose duty it shall be to examine such communications, and report upon the propriety of their presentation and reference to the Committee on Publication. Carried.

Dr. Smith, of N. Y., moved that a special committee be appointed to report at the next meeting of this Association, a classification of those diseases, which involve a derangement of the mental manifestations. Carried, and leave given the mover to appoint the committee, he being Chairman.

The Secretary read an invitation from Dr. Childs, of Boston, inviting the members of the Association to meet with the Massachusetts Medical Society, on the last Wednesday of May. Accepted.

Dr. Atlee, of Pa., moved that a copy of the Association's transactions be sent to the Epidemological Society, of London. Carried.

Dr. Gunn, of Mich., moved that any new Medical Society not heretofore represented in this Association, be required to transmit to the Secretary, with the credentials of its delegates, the evidence of its existence, capacity and good standing. Carried.

Dr. McGugin moved that a special committee be appointed to report on the subject of "Stomatitis Materna." Adopted, and Dr. McGugin appointed Chairman of such committee.

Dr. Bailey moved that Dr. N. S. Davis be requested to continue his observations on the subject of the changes produced in the composition and qualities of milk by pregnancy and menstruation; also, the best substitute for the mother's milk when weaning becomes necessary before the child is eighteen months old, and report at the next meeting of the Association. Carried.

Dr. Atlee, of Pa., moved that the thanks of the Association be tendered to all railroad companies who had furnished members with passes to this convention. Carrried.

Dr. Palmer, of Ill., called for some disposition to be made of the resolutions appended to his report on the organization of Medical Societies.

On motion of Dr. Atlee, they were referred, with the report, for publication.

Dr. Palmer, of Ill., moved that the Association tender a vote of thanks to the press of Detroit for the interest and attention given to their sessions in this city. Carried.

Dr. Palmer, of Ill., moved that the Committee on Registrations have leave now to present a partial report, which is hereby referred to the Committee of Publication. Carried.

Dr. Lindsley, on Tenn., offered the following:

WHEREAS, It is the object of this Association in the award of of its prizes for communications on subjects appertaining to medical science, to encourage the progress of the latter, and as this result cannot be better attained than through original investigation and discovery,

*Resolved*, That hereafter an annual prize of 00 be awarded for the best memoir or essay, founded on original investigation of the author, and in case of no memoir or essay being presented worthy of such award, the prize money to be appropriated towards the expense of publishing and illustrating such memoirs as may be subsequently deemed worthy of an award. Carried.

On motion the Association then adjourned to meet at Nashville, Tenn., on the first Tuesday of May, 1857.

